# The Relation Between Tobacco Tax Structure and Corruption in European Union Member States

**DOI:** 10.3390/ijerph16162842

**Published:** 2019-08-09

**Authors:** Ajay Shah, Katie Eminson, Ilze Bogdanovica, John Britton

**Affiliations:** UK Centre for Tobacco and Alcohol Studies, Division of Epidemiology and Public Health, University of Nottingham, Clinical Sciences Building, City Hospital, Nottingham NG5 1PB, UK

**Keywords:** tobacco tax, European Union, corruption

## Abstract

*Background*: Taxing tobacco products is one of the most effective tobacco control measures, and most countries apply a combination of specific taxes, which comprise a fixed amount per cigarette or gram of hand-rolling tobacco, and ad valorem taxes, which increase in proportion to the cost of the product. Since specific taxes reduce price differentials across tobacco product ranges while ad valorem taxes amplify them, we hypothesised that tobacco companies seeking to minimise the effect of tax increases on sales across a range of products will tend to favour, and hence lobby for, ad valorem rather than specific taxes; and that relatively corrupt governments would be more susceptible to such lobbying and hence, more likely to favour ad valorem taxes. *Methods*: We searched for cigarette tax data and Transparency International Corruption Perceptions Index (CPI) scores for current 28 EU Member States for the years 1995 to 2017/8. Trends in cigarette tax levels and the ratio of ad valorem to specific taxes at a national and mean EU level were analysed by visual inspection, the within-country relation between the ad valorem to specific tax ratio and CPI scores over time by time-series regression analysis, and at EU level, for which complete data were available from 1995 to 2017, using a multi-level regression model. *Results*: Within most Member States, the ad valorem to specific cigarette tax ratio declined over the study period and was not significantly associated with corruption score. However, at an aggregate EU-level, our multi-level model indicated that reduced corruption was associated with a significant increase in the ad valorem to specific cigarette tax ratio, by 0.04 (95% confidence interval: 0.003–0.077, *p* < 0.036) per unit increase in CPI score. *Conclusions*: The ratio of ad valorem to specific taxes declined in most EU Member States over the study period, with no evidence that those with higher levels of perceived corruption tended to favour ad valorem taxes.

## 1. Introduction

Taxing tobacco products is one of the most effective tobacco control measures [1,2,3], but can be undermined to a degree by the adoption of tax structures that apply disproportionately less to lower price products [4,5,6]. Tobacco products are typically subject to at least three taxes: the general sales taxes applied to most or all retail products in relation to their retail price; specific excise duties representing a fixed amount per cigarette (or gram of hand-rolling tobacco); and ad valorem excise duties applied in proportion to the value (usually the retail price) of the product. Increases in specific duties result in similar price increases across the full range of tobacco products, hence reducing relative price differences between them [1]. On the other hand, ad valorem taxes impose relatively small price increases on lower-price products, thus mitigating the effect of overall tax increases on the cost of tobacco use and sustaining lower-price products as a down-trading option for price-sensitive smokers [3,7,8].

In using tax as a health policy to reduce tobacco consumption it is, therefore, rational to favour specific over ad valorem taxes [7], since the resulting combination of higher prices and reduced price differentials between comparable products is more likely to encourage quitting. From a tobacco industry perspective, however, the converse approach, of ad valorem taxes which sustain relative price differentials, may be preferred [9]. European Union (EU) Member States are required by EU Directive 2011/64/EU to apply specified minimum duty levels on tobacco products [10], but the proportions of ad valorem and specific excise duty adopted by individual Member States vary considerably and the reasons for this variation are not clear.

One potential explanation is that tobacco companies lobby governments to adopt tax structures that minimise the impact on sales [11,12]. Previous research suggest that corruption is associated with tobacco control policy implementation in the EU [13]. Ad valorem tax-based systems typically result in lower prices, allowing tobacco to remain affordable and sustaining relative differences between economy and premium brand prices [14]. We, therefore, hypothesised that the governments that are relatively open to tobacco industry influence will be more likely to adopt ad valorem taxes, and since industry lobbying is likely to be more effective in corrupt administrations governments, that more corrupt countries will tend to apply higher ratios of ad valorem to specific tax than less corrupt countries. We have tested this hypothesis using cigarette tax data and corruption measures for EU Member States.

## 2. Methods

### 2.1. Data

Specific, ad valorem and overall minimum excise duty rates on manufactured cigarettes in EU Member States were collected from Excise Duty Tables prepared by the European Commission (EC) for the period 1995–2018, using data for July in each year if available and otherwise, using data from March 1995, June 1996, January 1997, September 1998, November 1999, May 2000, August 2002 and May 2004. In 2003, data were from April for Member States already acceded to the EU and July 2003 for new Member States that joined the EU in 2003. For the 13 countries that did not join the EU until after 1995, we contacted the relevant administrative offices to request excise duty data for the years between 1995 and joining the EU. All rates were converted into Euros (EUR) using the value of national currency in EUR on the 1st October or the first working day of October of the preceding year [15]. Between July 2003 and July 2005 in Bulgaria and Latvia, duty rates differed between filtered and un-filtered cigarettes; we use the filtered cigarette data. Between January 2004 and July 2008, Malta applied two duty rates according to cigarette circumference; we used the conventional king-size rate. Specific excise duties per 1000 cigarettes were gathered in EUR and as a percent of tax-included retail selling price (TIRSP) and weighted average price (WAP) pre and post-2011, respectively. Ad valorem excise duty rates were gathered as percent of TIRSP. The ratio of ad valorem to specific excise duty was then determined as the ratio of their proportions of retail price.

Corruption Perceptions Index (CPI) scores were obtained from the Transparency International website for individual countries from 1995, or the earliest available year after 1995, until 2017 [16]. The CPI measures the levels of corruption as perceived by experts and businesses [17,18]. Each year, the CPI uses a range of data sources that quantifies perception of corruption and is performed by a credible institution. The measure for each country result is obtained as an average of estimates from at least three sources. The CPI is based on perception as there is no direct indicator for quantifying corruption, and relates to various corrupt behaviours in the public sector, including bribery, diversion of public funds and use of public office for private gain. CPI scores before 2012 ranged from 0 (“highly corrupt”) to 10 (“very clean”), and from 2012 from 0 to 100; we, therefore, multiplied the early scores by 10 to correct for this change.

### 2.2. Analysis

We used basic descriptive statistical methods and parametric correlation to summarise and visually inspect trends in CPI and ad valorem to specific tax ratios within individual Member States and as EU mean values. Correlation analysis was carried out at a national and EU level and included all available data from 1995 to 2017. Time series analyses were done for individual Member State data and involved fitting an ordinary least squares time series regression model in RStudio (v3.4.2, Boston, MA, USA), assessing the relation between CPI score and ad valorem to specific excise duty ratio on an annual basis and including the date of introduction of Council Directive 2011/64/EU as a potential confounder. Individual country models were estimated via GLS by restricted maximum likelihood, and autocorrelation was checked using autocorrelation (ACF) and partial autocorrelation (PACF) plots. A mixed-effect regression model with random slopes was used to explore the relation at EU level (not for individual countries) between the ad valorem to specific duty ratio and CPI scores, and to explore the change in ratio over time, adjusting for implementation of EU Directive 2011/64/EU. A random sloped model was appropriate as it allowed each country to have a different slope. Stata v15.1 (StataCorp LLC, College Station, TX, USA) was used for the analysis.

## 3. Results

**Figure 1:**
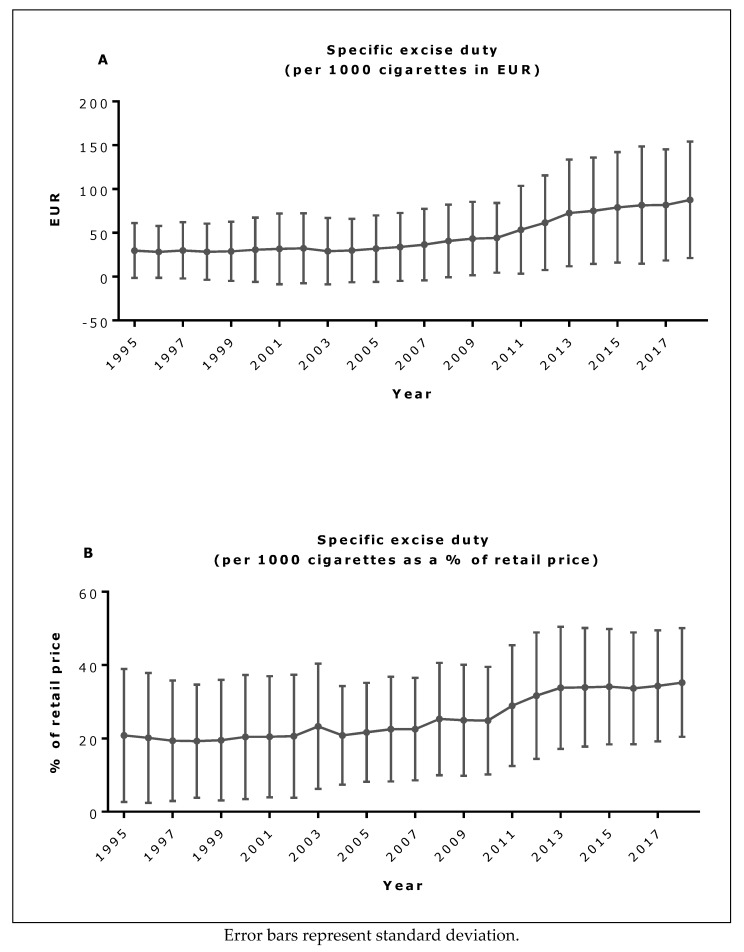
Mean EU specific excise duty in EUR (**A**) and as a percent of retail price (**B**).

**Figure 2:**
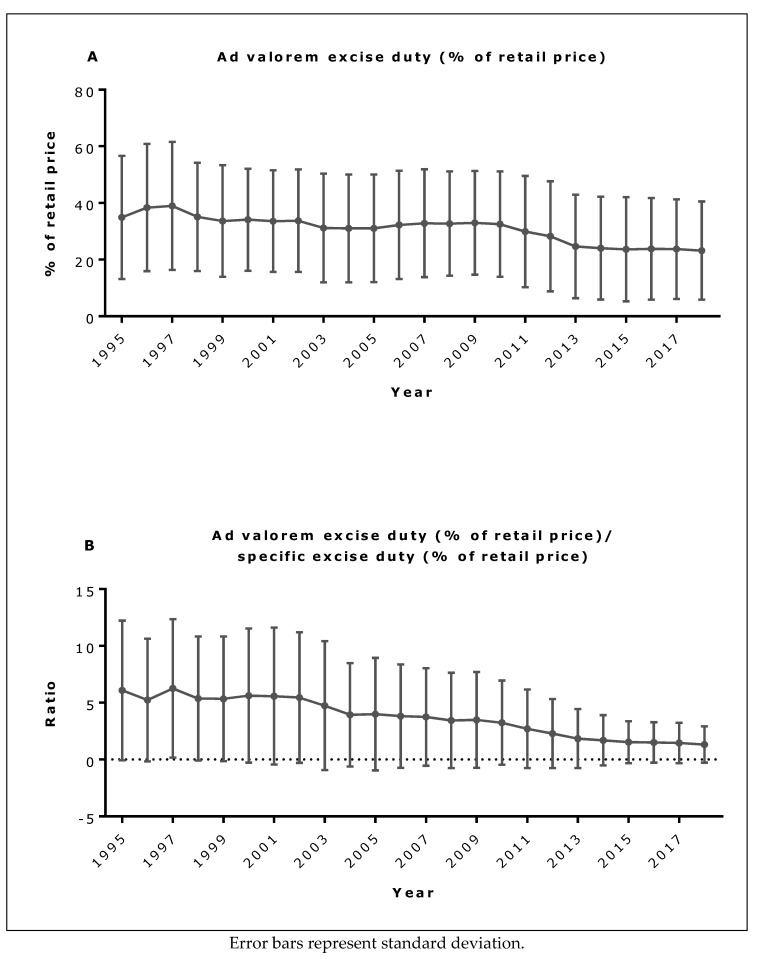
(**A**) Mean EU ad valorem excise duty as percentage of retail price and (**B**) mean EU ad valorem to specific excise duty ratio.

The mean level of specific excise duty EU-wide, both in EUR and as a percent of retail price, increased throughout the study period and particularly, after 2009 (Figure 1A,B). In contrast, mean ad valorem excise duty decreased over this period (Figure 2A), as did the ad valorem to specific excise duty ratio (Figure 2B). Ad valorem to specific excise duty ratios also fell over time in most Member States, and in almost half of all Member States (including Denmark, Greece, Netherlands, Sweden, Slovenia, Portugal, Slovakia, Malta, Lithuania, Estonia, Bulgaria and the UK), the ratio fell below unity, suggesting that specific excise duty became the more dominant duty in almost half of all Member States (data not shown).

**Figure 3:**
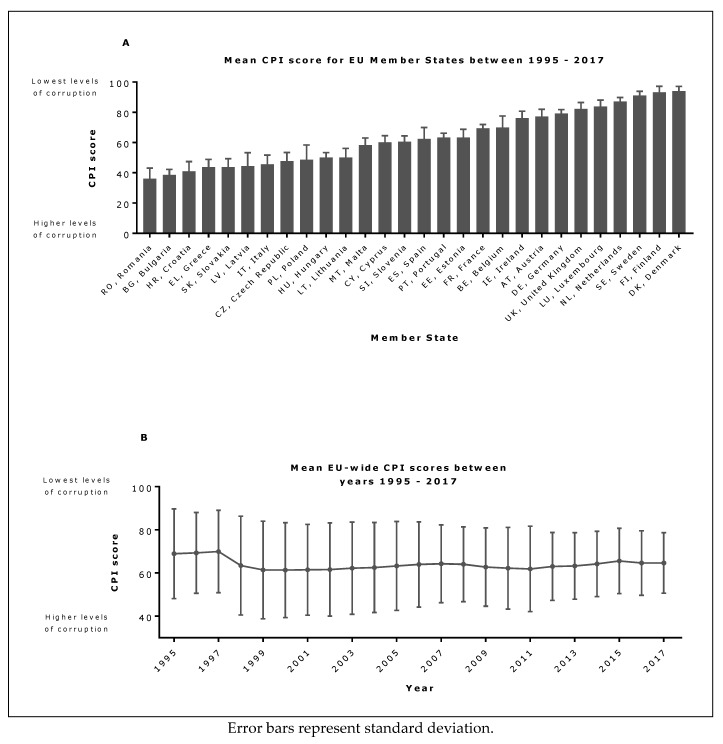
(**A**) Mean (1995–2017) Corruption Perceptions Index (CPI) scores for each EU Member State and (**B**) mean CPI scores EU-wide over time.

CPI scores varied substantially between Member States across an approximate 50-point range of mean values over the study period (Figure 3A), but showed relatively little change within Member States between 1995 and 2017, or as a mean EU-wide value (Figure 3B) over time.

Within almost all EU Member States, there was no significant correlation between CPI scores and the ad valorem to specific tax ratio each year (data not shown). Time series regression analysis also showed no significant association in most cases (Table 1), although the introduction of Directive 2011/64/EU had a significant impact in most countries, with the ratio falling significantly on an annual basis after 2011 (data not shown). At the EU level, there was a non-significant negative association (r = −0.06, *p* = 0.7750), suggesting no association between average corruption and average ad valorem to specific duty ratio (data from 1995 to 2017, Figure 4).

However, in a pooled analysis across all EU Member States, a mixed-effect regression model with random slopes demonstrated that the ad valorem to specific excise duty ratio decreased over time by 0.12 per year (95% Confidence Interval 0.168 to 0.073, *p* < 0.001) with an independently significant effect of corruption whereby for every unit increase in CPI score (i.e., decreasing corruption), the ad valorem to specific excise duty ratio increased by 0.04 (95% Confidence Interval 0.003 to 0.077, *p* < 0.036).

## 4. Discussion

This study demonstrates that, despite considerable between-country variations, the ratio of ad valorem to specific excise duty on tobacco has fallen in the EU over the past two decades, consistent with an EU tobacco taxation system which is moving towards specific excise duty and tobacco tax structures that favour health outcomes. However, this reduction was, if anything, more marked in the presence of higher levels of corruption. This means that our original hypothesis is rejected. There is no clear trend in association between mean ratio over time and mean CPI as among low-corruption countries, there are major differences in tax ratio, for example, between Denmark and Finland. This suggests that there is no uniform strategy for tobacco taxation and there may be alternative factors explaining approaches used by countries. Although flexibility in tax structures in individual Member States was constrained by the 2011 Directive which imposed minimum specific tax levels across the EU [10] and increased prices to varying degrees [19,20], the findings contradict our hypothesis that relatively corrupt Member States would be more likely to be persuaded by the tobacco industry to apply greater ad valorem excise duties.

Although our excise duty data were obtained from a range of EU and national sources and was subject to some variation in month of reporting and occasional missing data, to our knowledge our study draws on the most extensive excise duty dataset reported to date. We measured public sector corruption using the established and validated CPI measure [21], which combines data from a range of independent institutions specialising in governance and business climate analysis [22]. Since EU excise duty structures were harmonised by the 2011 Council Directive which imposed minimum levels of duty [10], we adjusted for this in our time-series analyses. We acknowledge, however, that our analysis does not allow for time lags inherent in the CPI scoring [22] and in implementation of the 2011 Directive. As perceived corruption levels tended to remain relatively constant at a country level over time, it is also possible that a lack of variation, rather than lack of underlying association, accounted for our null finding. It is also possible that CPI scores are a poor indicator of the extent of measures used by the tobacco industry to influence taxation policies. In the absence of a corruption measure that would allow us to investigate actual rather than perceived corruption levels, and investigation of time trends without any adjustments, we used CPI data to compare trends in corruption level over time. Since the scoring scale used in the CPI changed in 2012, we applied a simple multiplicative conversion to make measures more directly comparable, although we accept that we are unable to validate that comparability. The ecological analysis model we used is vulnerable to confounding [23], but our inspection of within-country trends (data not shown) indicated that within-country data were broadly consistent with the result of the ecological analysis. Although our model excludes illicit supply there are no *a priori* grounds to suspect that the associations described would be influenced by the size of the illicit market. We were unable to consider the impact of tax structure on actual price levels, contextualised with purchasing power parity. Our analysis was limited to EU Member States, which are subject to common rules on tobacco taxation which reduce diversity in the structures adopted; it is possible that our conclusions would be different if the study was repeated in a range of other countries. As our primary hypothesis was to explore the relation between tax structure and corruption, we did not investigate associations between tax structure and tobacco consumption. In addition, it has been shown that implementation of tobacco control is associated with corruption in the EU though after adjusting for a range of national-level country characteristics [13], which in this study were not considered. The importance of other country specific variables, such as per capita Gross Domestic Product and tobacco demand should be explored in relation to tobacco taxation and corruption in future studies.

The tobacco industry is adept at influencing tax levels imposed by governments, with the aim of reducing overall tax levels by lobbying for taxes which are socially regressive, unfair and likely to encourage illicit trade [24]. Ad valorem taxes are arguably less regressive than specific taxes, since they apply disproportionately less to the lower end of the tobacco price spectrum that is generally favoured by less affluent smokers. For this reason, tobacco companies with a broad portfolio of products are likely to favour ad valorem taxes, the effect of which on lower-price products can be further ameliorated by overshifting to more premium brands [6]. On the other hand, tobacco companies with predominantly premium brand product portfolios may favour specific taxes which have a relatively smaller effect on premium prices [24]. Further studies need to be done to explore whether this is the case and what lobbying strategies are used in the case of conflicting interests by various tobacco companies. In addition, in this study, we have focused on cigarette tax structure, however, there may be a relation between alternative tobacco products and their tax structure and corruption, which may warrant investigation.

## 5. Conclusions

The World Health Organisation advocates that over time, the ad valorem component of excise duty should be reduced and accompanied by proportionally larger increases in the specific component [7], with the result that specific duty becomes, proportionally, the largest component of the total excise tax on cigarettes. The World Health Organisation also recommends that in total, ad valorem and excise duties should together represent at least 70% of the retail price of the tobacco product [7]. In the present study, this threshold was passed by the combined excise duties in only the UK and Ireland (data not shown). It is, therefore, important that measures to increase tobacco prices continue, while further harmonisation between Member States may also help to reduce cross-border trade and illicit supply [25,26]. Further analysis is also required to determine the extent to which the different tobacco companies operating in the EU benefit from differences in excise duty structure, and how roll-your-own taxation has changed over time as it is frequently used as a substitute for manufactured cigarettes when prices increase.

## Figures and Tables

**Figure 4:**
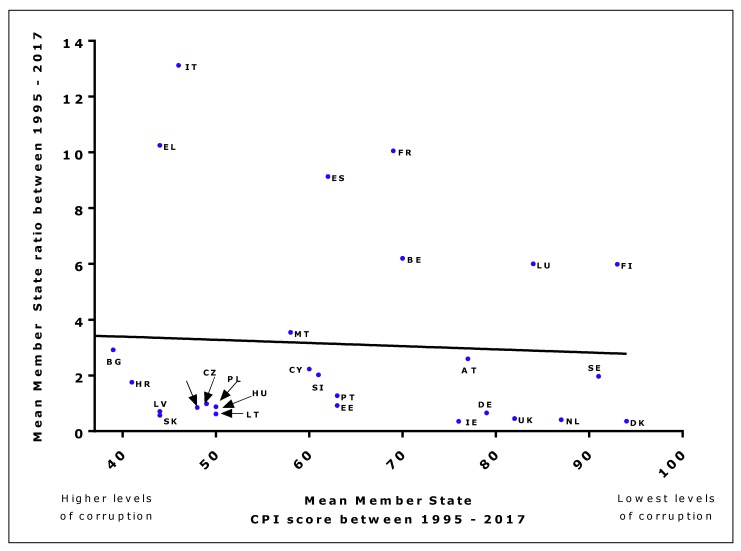
Mean CPI score and ad valorem to specific excise duty ratio across all EU Member States (data from 1995–2017).

**Table 1:** Time series regression estimates of coefficients for change in ad valorem to specific duty ratio per unit change in CPI score (data for Croatia insufficient for analysis).

Country	Coefficient	Standard Error	*p* Value
Austria	−0.020313	0.0077516	0.0173
Czech Republic	−0.0168546	0.00593855	0.0161
Poland	−0.0328598	−2.739512	0.0179
Slovakia	−0.0165421	0.00516629	0.0095
UK	−0.0041924	0.00117508	0.0021
Germany	0.00776925	0.00313698	0.0228
Bulgaria	0.489768	0.256643	0.0805
Cyprus	0.0135868	0.0114653	0.2634
Estonia	−0.0071129	0.0131971	0.5998
Finland	−0.033406	0.0205821	0.1211
Greece	0.159570	0.107282	0.1542
Hungary	0.0219810	0.0106499	0.0634
Ireland	0.00189133	0.00305938	0.5442
Italy	0.006323	0.0215210	0.7723
Latvia	−0.00154183	0.0124290	0.9037
Lithuania	−0.0036789	0.0113753	0.7525
Malta	0.041909	0.062992	0.5209
Netherlands	0.0075926	0.0040456	0.0760
Romania	−0.0177252	0.0350063	0.6226
Slovenia	−0.007171	0.0067619	0.3166
Belgium	0.033049	0.110575	0.7685
Denmark	0.013342	0.0137247	0.3432
Luxembourg	−0.33617	0.304799	0.2846
Portugal	−0.00621	0.098344	0.9503
Slovakia	−0.01654	0.0051662	0.0095
Spain	0.282165	0.059392	0.0002
Sweden	0.187649	0.101819	0.081
